# Tibetan Medicines and Tibetan Prescriptions for the Treatment of Diabetes Mellitus

**DOI:** 10.1155/2021/5532159

**Published:** 2021-05-17

**Authors:** Jie Gao, Lin Pan, Ruohong Bi, Yi Shi, Yunfeng Han, Xi Tang, Xianrong Lai

**Affiliations:** ^1^School of Pharmacy, Chengdu University of Traditional Chinese Medicine, Chengdu 611137, China; ^2^School of Ethnic Medicine, Chengdu University of Traditional Chinese Medicine, Chengdu 611137, China

## Abstract

Diabetes mellitus (DM) is one of the most serious diseases threatening human health and because of that, it is imperative to look for drugs to tackle it. The Tibetan medicine, a traditional medical system used in China, is currently being the focus of research towards the discovery of new effective drugs against several diseases. Based on the literature survey of Tibetan medicine monographs and drug standards, the Tibetan medicine, and Tibetan prescription used in the traditional Tibetan medical system, here, we summarise the methods indicated for DM treatment. In the Tibetan medical system, 56 types of Tibetan medicine and 25 Tibetan prescriptions were found for the treatment of DM. The most commonly used are Curcuma, Berberidis Cortex, and Carthami Flos. Their names, families, medicinal parts, phytochemical components, and pharmacological activities were described in detail in our research. These Tibetan medicines and prescriptions are valuable gifts from the Tibetan medicine to the world and may be the source of potential drugs for the treatment of DM. With the help of modern phytochemistry, pharmacology, metabonomics, and/or clinical trial methods, further research is needed to prove its medicinal value, identify bioactive components, elucidate potential mechanisms of action, and assess potential side effects or toxicity. This study provides the first available data compilation for the ethnic medical knowledge of Tibetan medicine for the treatment of DM, providing new ideas and sources for drugs against DM.

## 1. Introduction

Diabetes mellitus (DM) is a chronic disease that occurs when the pancreas does not produce enough insulin or the body cannot use the insulin produced effectively. Insulin is a hormone that regulates blood sugar. Hyperglycemia is a common result of uncontrolled DM. Over time, this disease will cause serious damage to many systems of the human body, especially nerves and blood vessels. According to the World Health Organization (WHO), DM will become the seventh leading cause of death by 2030. The International Diabetes Federation (IDF) has released the 9th edition of the global DM map [1] and, according to it, in 2019, about 463 million adults aged 20–79 years suffered from DM (1 out of 11 people is a DM patient); it is estimated that, by 2030, the number of DM patients will reach 578.4 million and, by 2045, 7,002 million (http://www.diabetesatlas.org/). The top three countries with higher DM incidence are China, India, and the United States of America. Amongst them, China has the highest burden of DM with 116.4 million patients. According to the WHO report, DM may damage the heart, blood vessels, eyes, kidneys, and nerves, which may lead to other complications including diabetic angiopathy, diabetic nephropathy (DN), and diabetic neuropathy. Therefore, it is necessary to explore effective drug methods for the treatment of DM. Currently, the control and treatment of DM and its complications mainly rely on chemical or biochemical agents [2, 3]. The Tibetan medicine has accumulated experience in the treatment of DM leading to lesser side effects and lower cost and improving the overall symptoms.

**Figure 1:**
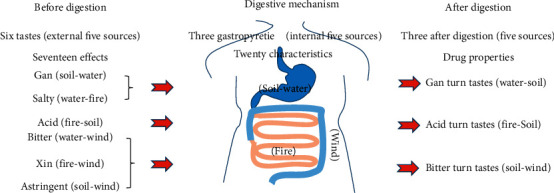
Schematic diagram of transforming Six Tastes into Three After Digestion under the action of “Three Gastropyretie” in Tibetan medicine.

DM belongs to the category of “jingnisaku disease” (Chinese direct translation: frequent urination) in Tibetan medicine; “jingni” means frequent urination and “saku” means turbid liquid that consumes physical energy. It is known though that frequent urination, turbid urine, and physical consumption are characteristics of this disease. Tibetan medicine believes that DM has both internal and external causes and that there is an important relationship between “Three Gastropyretie” (Figure 1) and metabolic syndrome. Tibetan medicine vividly and meticulously describes the process of digestion, absorption, and transformation of food in “Six Tastes,” “Three Gastropyretie,” and “Three After Digestion.” Amongst them, “Three Gastropyretie” is mainly distributed in the digestive tract and plays an important role in food digestion and in maintaining a balanced immune function. In addition, the Tibetan medicine clearly pointed out that excessive Bad-kan caused by the “Three Gastropyretie” disorder is the pathological basis of “jingnisaku disease” (belonging to the category of metabolic syndrome and DM) [4]. The ancient book of Tibetan medicine *Rgyud bzhi* explains the outer edge of the “frequent urination syndrome” in this way: “due to eating salty, sweet, cold, or heavy diet, living in a humid place for a long time, growing Bad-kan size, leaking into the bladder, producing body fluids, and causing disease.” It can be seen that the outer edge of the “jingnisaku disease” is mainly caused by improper diet, the environment, and other factors, resulting in the imbalance of the “three causes” [5–8]. In the traditional Tibetan medical system, diabetic diseases are divided into three types, namely, rLung enuresis, Bad-kan enuresis, and mKhris-pa enuresis. In the *Rgyud bzhi*, the symptoms are subdivided into three categories according to the clinical manifestations: six “rLung” types, ten “mKhris-pa” types, and 20 “Bad-kan” types.

According to the Tibetan medicine, the human body is as a whole connected by various parts and, according to the *Blue Glaze*, “there are four or five major veins on the human body named as dependent arteries.” According to this principle, the body functions are connected with each other and restricting parts of the body where diseases, “essence,” and filth are located. The aforementioned four dependent veins take their respective positions as the axis, and shoot branches and subbranches are from the left, right, and middle parts of the human body; they are distributed across all parts of the human body contacting tissues and organs, so that the three genes, seven matrices, and three filth of the human body can operate normally. The “holistic view” of the Tibetan medicine approaches the person as a whole, and consequently, the occurrence and development of DM can lead to diseases in other parts of the body, in a later stage. Therefore, while treating the “frequent urination” symptoms, we should pay attention to the Tibetan medicine's holistic view of the disease and focus on the balance between the three genes. Tibetan medicine believes that the external cause of “jingnisaku disease” is mainly caused by the imbalance of “three causes” caused by improper diet and environmental conditions. Therefore, a series of treatment methods based on diet, daily life, and drugs against the “jingnisaku disease” are put forward.

**Figure 2:**
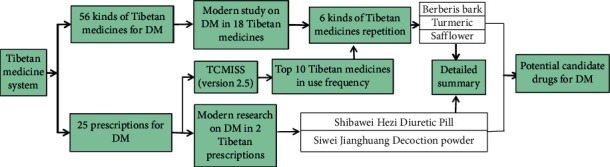
Technical road map of this paper.

According to the statistics in the *Dictionary of Chinese Ethnic Medicine* [9], there are 3105 different types of natural drugs used in the Tibetan medical system, including 2644 plants, 321 animals, and 140 minerals. Tibetan medicine has accumulated experience in the treatment of various diseases, most particularly against chronic diseases such as DM, hepatitis, high altitude polycythemia, gastritis, stroke, cholecystitis, and rheumatism. It is worth noting that the Tibetan medicine has been extensively used in the treatment of DM. However, most of these records are scattered and there is a lack of systematic summaries and inductions. This paper summarises two groups of research: one is data from Tibetan medicine to treat DM from *Jing Zhu Materia Medica*, *Dictionary of Chinese Ethnic Medicine, Drug Standards of Tibetan Medicine,* and *Blue Glaze*; the other is prescriptions for treating DM from *Drug Standards of Tibetan Medicine*, *Treasure House of Tibetan Medicine Prescriptions*, and *Chinese Materia Medica Tibetan Medicine Roll* (Figure 2).

## 2. Materials and Methods

We have manually searched ten Tibetan medicine monographs and drug standards, such as *Jing Zhu Materia Medica, Dictionary of Chinese Ethnic Medicine, Drug Standards of Tibetan Medicine, Rgyud bzhi, Blue Glaze*, and *Treasure House of Tibetan Medicine Prescriptions*. To obtain information about Tibetan medicines and prescriptions used in the treatment of DM, names of species, families, Tibetan names, and medicinal parts have been recorded. The names of these Tibetan medicines have been published in the Flora of China (http://frps.eflora.cn/) and the database was verified according to their Chinese and Latin names. In order to understand the most commonly used DM Tibetan medicine, a data mining method was used to obtain its usage frequency. By using the Traditional Chinese Medicine Inheritance Support System (version 2.5) [10], all the collected Tibetan medicines were manually input into the TCMISS software. In addition, we searched Chinese online databases (such as CNKI, WanFang, and VIP) and international databases (such as GeenMedical and sci-hub) and used their dialect, English, or Latin names as keywords to obtain the active ingredients and biological or pharmacological effects.

## 3. Results

### 3.1. Literature Research Results of Tibetan Medicines against DM

**Figure 3:**
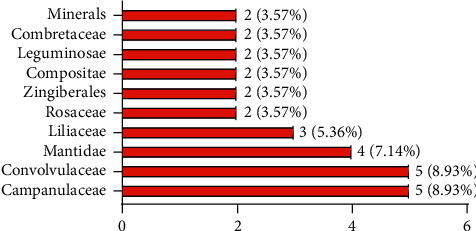
The most common families and genera of Tibetan medicine in the treatment of DM.

This paper records the application of 56 Tibetan medicines and 25 Tibetan prescriptions belonging to the traditional Tibetan medical system, towards a treatment against DM. Amongst the 56 Tibetan medicines, 20 have been used in modern research for the treatment of DM and diabetic complications. The scientific names, Chinese names, Tibetan names, families, medicinal parts, therapeutic diseases, and reported biological activities of these 20 Tibetan medicines are shown in Table 1. These drugs are distributed in 31 families and genera. The most common families are Campanulaceae (8.9%), Convolvulaceae (8.9%), Mantis family (7.1%), and Liliaceae (5.3%) (Figure 3). Amongst the 56 kinds of Tibetan medicine for DM, five are still not studied by modern research methods, and 31 have not been used in modern research for the treatment of DM. Therefore, in order to make better and safer use of these Tibetan medicines, it is necessary to conduct a more in-depth and systematic study.

It can be seen from Table 1 that the biological activity of a small number of Tibetan medicines is not focused on a single natural product, for example, the angelica extract, *Xanthium sibiricum* extract, *Amomum kravanh* oil, and total flavonoids of *Xanthium sibiricum*. Additionally, only a small number of Tibetan medicines have a clear mechanism of action; there is only a whole concept. We believe that a more extensive research on specific Tibetan traditional medicines could make the overall medical system more factual, reliable, and safe. Through the analysis of the table, we have selected three Tibetan medicines used for the treatment of DM for a detailed analysis. Moreover, modern research has proved that all three can be used in DM, DR, and DN.

#### 3.1.1. Berberidis Cortex

*Berberis kansuensis* Schneid (Latin name of original plant), also known as སྐྱེར་པ། (Tibetan name), Xiao Bopi (Chinese name), or Berberidis Cortex (Latin name of medicinal materials), is the dry endothelium of many Berberis plants in Berberidaceae (such as *Berberis dictyophylla* Franch.). The ancient Tibetan medicine literature *Blue Glaze* (p. 382) records “the frequent micturition caused by Bad-kan transformation and its treatment method (…), Berberidis Cortex (…), any kind of fried soup, with honey, orally” [8]. According to ancient Tibetan medicine books, Berberidis Cortex can treat DM.

The main components of Berberidis Cortex are berberine, magnolia alkaloid, palmatine, jatrorrhizine, and berbamine [53, 54]. Berberine can significantly reduce blood glucose and blood lipids and be used as antibacterial, antiviral, and antitumoral. Zhang found that Berberidis Cortex of Tibetan medicine had an obvious hypoglycaemic effect on alloxan-induced diabetic mice and no effect on blood glucose in normal mice [55]. Labaciren et al. [22] and Zhou et al. [56] explored the use of Berberidis Cortex for DR from a theoretical point of view and medication experience in Tibetan medicine. Zhou et al. [56] concluded that the mechanism of Berberidis Cortex extract in the prevention and treatment of DR in db/db mice would be related to the inhibition of PKC-*β*. Yue et al. [20] found that Berberidis Cortex has a protective effect on the retina of diabetic rats and its mechanism was related to the overall multipoint regulation of PKC-*β*, HIF-1*α,* and VEGF expression in the retina. Berberine and berbamine are the main components in Berberidis Cortex. Modern studies have shown that berberine and berbamine can be used for DR. Berberine regulates the expression of P-TEFb (CDK9 and CyclinT1) in the retina, which was proposed to be one of the mechanisms of berberine against DR. Berberine can be considered as one of the direct effective substances regulating the retinal vascular endothelial homeostasis. Berbamine blocks voltage-dependent and receptor-dependent calcium channels and protects retinal cells by inhibiting the calcium influx, similar to verapamil. Based on metabonomics, AI et al. explored the protective mechanism of Berberidis Cortex on STZ-induced DN rats and found specific serum metabolic biomarkers. Berberine improved the pathological changes and pharmacodynamic indexes of DN by increasing the content of ornithine in the serum of DN rats and participating in the metabolism of arginine and proline [57].

In conclusion, the role of Berberidis Cortex against DM is not only based on the Tibetan medicine but also supported by modern research. However, its mechanisms of action remain unclear.

#### 3.1.2. Curcumae Longae Rhizoma

*Curcuma longa* L. (Latin name of original plant) is also known as ཡུང་བ (Tibetan name), Jiang Huang (Chinese name), or Curcumae Longae Rhizoma (Latin name of medicinal materials). Ancient Tibetan medicine recorded the treatment of DM with Curcumae Longae Rhizoma. In *Rgyud bzhi*, the frequent urination syndrome is treated with “Curcumae Longae Rhizoma officinalis.” In addition, the Tibetan medical classics *Rgyud bzhi* describes that the treatment against DM is made up of Berberidis Cortex, Curcumae Longae Rhizoma, Phyllanthi Fructus, and Tribuli Fructus.

The main chemical components of Curcumae Longae Rhizoma are its volatile essential oil and curcumin, which has anti-inflammatory, anticancer, antioxidation, and lipid-lowering effects. It can also protect the kidney, inhibit liver and pulmonary fibrosis, help in repairing muscle damage, treat cataracts, and resist parasitic diseases. Modern research on Curcumae Longae Rhizoma in DM and its complications has been reported. Gunnink et al. [16] found that curcumin can inhibit directly the glucose transport in adipocytes. He Qiong et al. [17] concluded that curcumin was able to inhibit the expression of E26 transcription factor-1 (Ets-1) in retina of diabetic rats and Mao Xinbang et al. [18] explored the role of hypoxia-inducible factor-1*α* (HIF-1*α*) in DR and the effect of curcumin in its expression. It was found that curcumin could delay the development of DR by downregulating HIF-1*α*. Gu Xuejing et al. [58] studied the effect and mechanisms of curcumin on retinal Müller cells of early diabetic rats and found that curcumin upregulated the expression of GS, cleared retinal glutamate, and protected Müller cells. Its mechanism of action would be related to curcumin inhibiting the oxidative stress of the diabetic retina. Curcumin is also known to inhibit angiogenesis and to reduce the expression of VEGF, reducing renal sclerosis stress injuries and fibrosis progression of DKD rats by regulating the JNK pathway of rats [19].

In summary, Curcumae Longae Rhizoma has been studied in modern research against DM, DR, DN, and other diabetic complications such as diabetic hepatopathy and cardiomyopathy.

#### 3.1.3. Carthami Flos

*Carthamus tinctorius* L. (Latin name of original plant), also known as གུར་ཀུམ (Tibetan name), Hong Hua (Chinese name), or Carthami Flos (Latin name of medicinal materials), is a common plant used in Tibetan medicine. It is widely planted in Henan, Sichuan, Xinjiang, and Zhejiang provinces in China. Carthami Flos is one of the most commonly used Tibetan medicines to treat DM. In this paper, 25 Tibetan prescriptions of DM were collected and Carthami Flos appeared 13 times. Carthami Flos has been also used in the treatment of dysmenorrhoea, dystocia, trauma, and blood stasis in Tibetan outpatients for thousands of years.

So far, many chemical constituents have been isolated from Carthami Flos, such as hydroxysafflor yellow A, Carthami Flos yellow A, luteolin, kaempferol, and adenosine [59]. Hydroxysafflor yellow A is its main bioactive component and often used as a marker for quality control in the pharmaceutical industry. Xiong Liping et al. [46] have shown that Carthami Flos yellow injection could be used to treat early-stage type 2 DR and to improve the imbalance of VEGF and ES secretion. Chen Yan et al. [48] showed that an injection of Carthami Flos had a positive effect on improving serum-related indicators and fundus blood flow in patients with DR, so its clinical application value in patients with DR is relatively high. Zhang Shuai et al. [49] showed that safflower yellow reduced the contents of VEGF and PDGF in retina of diabetic rats, inhibiting the formation of new blood vessels and delaying DR. Gao Yan [60] and other researchers have found that safflower yellow presented anti-inflammatory activity in DN by downregulating the expression of caspase-1 and NLRP3 mRNA. The research on Carthami Flos against DM and its complications is mainly focused on safflower yellow.

### 3.2. Literature Research on the Treatment of DM with Tibetan Prescription

**Figure 4:**
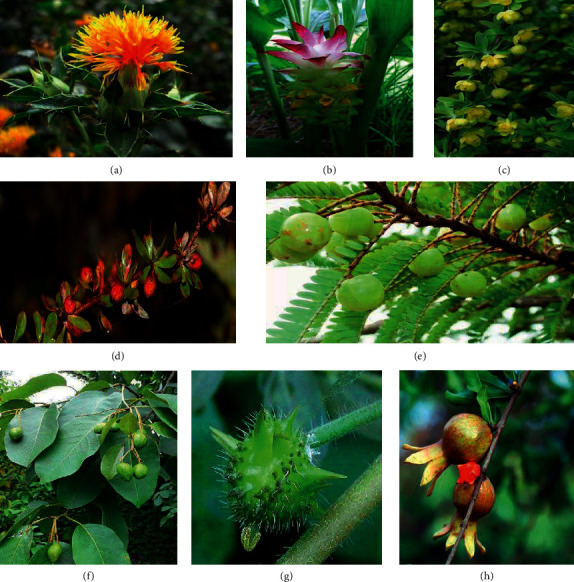
The picture of common Tibetan medicine in Tibetan prescriptions. (a) *Carthamus tinctorius* L. (b) *Curcuma longa* L. (c) *Berberis dictyophylla* Franch. (d) *Berberis dictyophylla* Franch. (e) *Phyllanthus emblica* L. (f) *Terminalia chebula* Retz. (j) *Tribulus terrestris* Linnaeus. (h) *Punica granatum* L.

In order to understand the most commonly used Tibetan medicine for DM, TCMISS software was used for data mining to obtain the frequency of Tibetan medicine in DM prescriptions (Figure 4). Twenty-five diabetic prescriptions were collected from the *Treasure House of Tibetan Medicine Prescriptions*. Amongst the prescriptions for DM, the top ten Tibetan medicines were Phyllanthi Fructus (15 times), bear gall (15 times), Chebulae Fructus (14 times), Curcumae Longae Rhizoma (13 times), Berberidis Cortex (13 times), Tribuli Fructus (13 times), Carthami Flos (13 times), *Amomum kravanh* (11 times), Granati Pericarpium (eight times), and *Terminalia bellirica* (5 times). In addition, nine types have been researched in the modern days against DM (Table 1). Amongst the ten Tibetan medicines, six (Phyllanthi Fructus, Chebulae Fructus, Curcumae Longae Rhizoma, Berberidis Cortex, Tribuli Fructus, and *Amomum kravanh*) have already been identified as potential treatments against DM. Additionally, amongst the 25 Tibetan prescriptions collected in this paper, two have already found significance in DM-related modern research. A detailed summary of these two Tibetan prescriptions is presented below.

#### 3.2.1. Shibawei Hezi Diuretic Pill

Shibawei Hezi diuretic pill is a Tibetan medicine named jinniarujiujieribu. The prescription is derived from the supplement to Tibetan medical formula and included in the Ministry of Health drug standard Tibetan medicine (No. WS_3_-BC-0182-95). It is a compound preparation prepared by the traditional processing of 18 types of traditional Tibetan medicinal materials such as Phyllanthi Fructus, Berberidis Cortex, Chebulae Fructus, and Tribuli Fructus. The Shibawei Hezi diuretic pill can be used in kidney disease, frequent urination, low back pain, spermatorrhea, DM, and other diseases. It is one of the most commonly used hypoglycaemic drugs in Tibetan medicine [61].

Shibawei Hezi diuretic pill has been proven to treat DM in recent studies. Suonanco et al. Shibawei Hezi diuretic pill could achieve a more significant clinical treatment for DM not only by controlling the overall blood glucose level of patients but also by improving the occurrence of adverse reactions [62]. Liang Peiyu et al. [63] found that Shibawei Hezi diuretic pill had a hypoglycaemic effect related to the repair of islet B cells. In 2019, Liang Peiyu et al. [61] concluded that Shibawei Hezi diuretic pill had a good hypoglycaemic effect by promoting the body's uptake and utilisation of glucose, improving antioxidant capacity, and reducing the damage in the pancreatic island. The prescription of Shibawei Hezi diuretic pill is as follows: 200 g of Chebulae Fructus, 40 g of Amomi Fructus Rotundus, 80 g of kaempferol leaves, 80 g of madder, 100 g of Curcumae Longae Rhizoma, 100 g of Tribuli Fructus, 80 g of corydalis saxicola, 2 g of bear bile, 100 g of Carthami Flos, 60 g of rock extract, 80 g of purple grass mushroom, 150 g of Phyllanthi Fructus, 100 g of Berberidis Cortex, and 100 g of golden peony of the 18 drugs.

To sum up, recent studies have already reported the effect that Shibawei Hezi diuretic pill has in treating DM. Only more research will tell if this natural medicine can be adapted to a completely safe and controlled DM drug.

#### 3.2.2. Siwei Jianghuang Decoction Powder

Siwei Jianghuang Decoction powder is a Tibetan medicine named Yong wa Xi Tang. The prescription is derived from the *Blue Glaze*, with only the name of each medicine and no specific drug dosage recorded. In *the Standard of Tibetan Medicine* compiled by the Tibet Health Bureau, it is recorded a soup powder made of four types of traditional Chinese and Tibetan medicinal materials, including Curcumae Longae Rhizoma 100 g, Berberidis Cortex 140 g, Phyllanthi Fructus 120 g, and Tribuli Fructus 140 g. Siwei Jianghuang Decoction powder can be used in the treatment of urethritis, frequent urination, urinary urgency, and other diseases. It is one of the most commonly used drugs for treating frequent urination in Tibetan medicine.

Tong Dong et al. [64] found that Siwei Jianghuang Decoction powder is effective in the treatment of DN by regulating the overexpression of HIF-1*α*, VEGF, and TGF-*β*1. A preliminary study on the Tibetan medicine Siwei Jianghuang formula in a STZ-induced DN rat model found that the optimal matching dose of Curcumae Longae Rhizoma: Berberidis Cortex: Phyllanthi Fructus: Tribuli Fructus should be 1 : 2 : 1 : 2 [65]. Tan Juan et al. [66] found that Siwei Jianghuang Decoction powder alleviated kidney injury in DN rats by activating the PI3K/Akt signalling pathway and reducing the podocyte injury. The prescription of Siwei Jianghuang Decoction powder is reported as follows: Curcumae Longae Rhizoma 100 g, Berberidis Cortex 140 g, Phyllanthi Fructus 120 g, and Tribuli Fructus 140 g. Amongst these four medicines, Curcumae Longae Rhizoma, Tribuli Fructus, Phyllanthi Fructus, and Berberidis Cortex have been recently studied as extracts against DM, as shown in Table 1. Thus, this also confirms that Siwei Jianghuang Decoction powder is effective in the treatment of DM.

## 4. Deficiencies and Prospects

Although Tibetan medicine has contributed positively to the treatment of several diseases, the potential toxicity associated with traditional medicines should not be overlooked. For example, in 1998, Lin Banghe and colleagues found that the Carthami Flos that decoction contained crude drug in the concentration of 0.24, 0.48, and 0.96 g/kg was force-fed to pregnant rats. It was concluded that that extract was toxic to the pregnant rat and its embryo and led to abortion, weight loss, increase of kidney weight index, increase of embryo mortality, and intrauterine growth retardation (IUGR) [67]. Zhao Yunlong et al. [68] also confirmed the toxic effect of Carthami Flos normal pregnant mice [69]. Although Tibetan medicine has a good curative effect in the treatment of DM, its potential toxicity should be further studied.

In this paper, only a few of the Tibetan medicine for DM have been studied recently and many lack a theoretical basis for their biological activities. Nowadays, the detection standards for the Tibetan medicine are still not ideal; there are only 136 (4%) Tibetan medicines recorded in the *Standards issued by the Ministry of Tibetan Medicine*. The performance of the records is very few and generally there is no TLC identification or content identification.

Mineral Tibetan medicine is the fifth most commonly used medicine in the treatment of DM, accounting for 43 prescriptions (21.5%) of the total number of records [70]. However, the safety of mineral drugs is still challenging, as the Tibetan medicine has a unique method for its use. *Jing Zhu Materia Medica* is a representative book recording the adverse reactions of mineral drugs. The book not only emphasises the properties of Tibetan drugs but also recognises their toxicity. For example, *Me Na Ba Bu Mu* referred that “gold tastes astringent, tastes bitter, cool, detoxification, and longevity, but it is toxic and can be sterilised” and in *Gan Lu Xiao Deng*, we can find that “lime is not cooked into medicine, such as poison into medicine, and can kill stomach fire and block blood vessels, swelling, iron scale disease, and ascites xiphoid swelling.” Therefore, it is believed that Tibetan medicine believes that some poisonous medicinal materials should be further processed. *Xia Da Mu* referred that “lime water solution precipitation, such as yogurt, with silk or fine cloth filter, the size of flour such as sesame.” More than 90% of mineral drugs in Tibetan medicine are processed under different “processing properties (heat, cold, and flat).” *Yuewang Medicine Diagnosis* proposed that “cold water stone mainly for the treatment of tummy, dispel cold, liver and blood gall disease, harmful.” There are also ancient records that recorded that “no matter what kind of cold water stone you take, you have to detoxify it. It's a stupid way to use it raw.” This shows that the Tibetan medicine not only discovered the therapeutic effect of a mineral medicine but also recognised its harmful side effects. Therefore, it was necessary to eliminate or reduce the toxicity of drugs prior to their clinical application.

In conclusion, we find value in the Tibetan medicines against DM, although its potential toxicity should not be overlooked. We consider that dosage is particularly important to prevent drug-induced injuries. It is necessary to standardise Tibetan medicine preparations and Tibetan medicines, increase research funding, and seek more scientific and experimental basis through research on toxicity, pharmacokinetics, safety, and effectiveness. Only then the Tibetan medicine can be used with confidence in Western civilisations.

## 5. Discussion

Tibetan medicine is an important part of the world's traditional medical system, which has accumulated a lot of experience particularly in the treatment of chronic diseases. These medicines usually present small side effects, are affordable, and can effectively improve symptoms related to diseases. Many commonly used drugs in modern medicine have been generated directly or indirectly from natural drugs, such as artemisinin and paclitaxel. In this paper, a total of 56 types of Tibetan medicine and 25 Tibetan prescriptions have been collected. The results show that Tibetan medicine is mainly distributed across 31 families and genera, with Campanulaceae being the most common.

In addition, it is necessary to point out the gaps and limitations in the current research on Tibetan medicines. Amongst the 56 types of Tibetan medicine, 18 have been used in modern research against DM and diabetic complications. Here, we propose a further focus on researching these last 18 types of medicines using a multidisciplinary approach, in order to reveal their modes of action, elucidate their absorption, distribution, metabolism, and excretion pathways, and evaluate their potential toxicity.

The purification and extraction of single drugs and the elucidation of their mechanism of action are additional problems to be addressed. Extraction methods used included preleaching and liquid ammonia pretreatment [71]. One of the biggest differences between Tibetan medicine and Western medicine is the complexity and diversity of the Tibetan medicine as it relies on the joint action of multiple components. However, it is recognised that the action of a single component of any Western medicine has a more precise mechanism and safety profile.

Here, we propose that the 31 types of Tibetan medicine found should be used as an extract in preliminary research before the active components are extracted; the concentration of active components can be compared with previous research on single natural products against DM in plants from the same genera, for example. For the five types of Tibetan medicines without additional modern research, an initial study on their extracts' activity against DM should be carried out, matching the theoretical basis of Tibetan medicine with benchwork.

A special mention should be made to several active natural products already extracted from Tibetan medicines, such as safflower yellow, curcumin, berberine, and ursodeoxycholic acid, which may be promising candidates for the treatment of DM.

Switching to nonpharmacological options recorded in documents of Tibetan medicine, it is important to focus on the “Jingnisaku disease” etiology: diet and improper living, which lead to the imbalance of the “three factors.” A series of treatment methods related to food are already described in Chinese medicine such as mulberry [72] and tea [73]. Wei Helin et al. proposed that mulberry is a promising therapeutic agent for the treatment of DM.

In conclusion, this study provides the first compilation of data in ethnomedicinal knowledge of Tibetan medicine and Tibetan prescriptions in the treatment of DM. The medicinal species that are highly reported in the documents should contain valuable active compounds against DM. In order to better develop and utilise these traditional Tibetan medicines, an effort should be made in order to evaluate their biological activities *in vitro* and *in vivo*, identifying bioactive components, elucidating their mechanism of action, and clarifying possible effects or toxicity by using a range of methods such as pharmacological, phytochemical, metabonomics, and/or clinical trials.

## Figures and Tables

**Table 1:** Tibetan medicine for DM in modern research (the order of Tibetan medicine names is from high to low according to the frequency of use).

No.	Latin name	Chinese name	Tibetan name	Family	Medication part	Study on the treatment of DM and its complications	Reported biological activities associated with DM
(1)	*Phyllanthus emblica* L.	Yu Ganzi	སྦྱུ་རུ་ར	Euphorbiaceae	Fruit	Gallic acid (GA) can downregulate the gene expression of NLRP3 and TXNIP [11] and inhibit the apoptosis of islet beta cells induced by high glucose; the ethanol extract of *Phyllanthus emblica* can enhance the expression of glut4mrna in skeletal muscle, thus improving insulin resistance and treating DM [12, 13].	Gallic acid
(2)	*Terminalia chebula* Retz.	He Zi	ཨ་རུ་ར	Junzi family	Fruit	In diabetic rats, chebula extract can reduce the damage caused by the structural and functional changes of mitochondria in the process of oxidative stress and has antioxidant activity and thus antidiabetic activity [14, 15].	*Terminalia chebula* extract
(3)	*Curcuma longa* L.	Jiang Huang	ཡུང་བ	Zingiberaceae	Rhizome	Curcumin can directly inhibit glucose transport in adipocytes [16]. Curcumin can inhibit the expression of Ets-1 and HIF-1a in the retina of diabetic rats and inhibit oxidative stress in the retina of diabetic rats, so as to alleviate the diabetic retinopathy (DR) process [17, 18]. Curcumin may protect the renal function of DKD rats by regulating JNK pathway and reducing the progression of renal sclerosis stress injury and fibrosis in DKD rats [19].	Curcumin
(4)	*Berberis kansuensis* Schneid	Xiao Bopi	སྐྱེར་པ།	Berberidaceae	Endothelium	Berberis Cortex has a protective effect on retina of diabetic rats [20, 21]. Berbamine can block the voltage-dependent calcium channel and receptor-dependent calcium channel. It can be considered that berbamine may be one of the direct therapeutic substances regulating the retinal vascular endothelial homeostasis in Tibetan Cortex Berberis bark [22]. Berberis bark can improve the pathological changes and pharmacodynamic indexes of DN [22].	Berberine and berbamine
(5)	*Lycium chinense* Miller	Gou Qi	རདྲེ་ཆོར་མ།	Solanaceae	Fruit	Lycium barbarum polysaccharide can significantly reduce blood glucose and increase insulin index in diabetic patients [23]. Lycium barbarum polysaccharide can effectively prevent and treat DR by increasing the expression of bcl-2 mRNA and protein, reducing the expression of Caspase-3, BAX mRNA, and protein, and reducing the apoptosis of retinal ganglion cells [24, 25]. Lycium barbarum polysaccharide has a protective effect on kidney tissue of type 2 diabetic mice, and it is related to the upregulation of PPAR-*γ* protein expression [26].	Lycium barbarum polysaccharides
(6)	*Tribulus terrestris* Linn.	Ji Li	གཟེ་མ	Tribulaceae	Fruit	The effect of *Tribulus terrestris* extract may be related to the decrease of TLR2 and TLR4 mRNA content and the inhibition of inflammation [27]. *Tribulus terrestris* saponin has an inhibitory effect on intestinal *α*-glucosidase and can reduce the increase in postprandial blood glucose level in normal and type 2 diabetic rats [28].	Caltrop saponin
(7)	*Amomum kravanh* Pierre ex Gagnep.	Bai Doukou	སུག་སྨེལ	Zingiberaceae	Fruit	The volatile oil of cardamom Bunge upregulates the expression of MMP-2, TGF-*β*1, and IGF-2, thus changing the pathological state of DM [29].	Volatile oil of cardamom bungeanum
(8)	*Codonopsis pilosula* (Franch.) Nannf.	Dang Shen	ཀླུ་བདུད་རྡོ་རྗེ།	Platycodonaceae	Root	*Codonopsis pilosula* polysaccharide can reduce blood glucose in diabetic mice and improve insulin resistance in mice [30].	*Codonopsis pilosula* polysaccharide
(9)	*Rosa laevigata* Michx.	Jing Yinzi		Rosaceae	Fruit	*Rosa laevigata* can reduce the blood glucose level of DM and has preventive and therapeutic effects on DN and liver disease. It can delay the development of diabetic cataract [31–34].	
(10)	*Cuscuta chinensis* Lam.	Tu Sizi	སྦྲུལ་ཞགས།	Convolvulaceae	Seed	Cuscuta polysaccharide can improve the metabolism of lipopolysaccharide in experimental diabetic rats [35].	Dodder polysaccharide
(11)	*Asparagus cochinchinensis* (Lour.) Merr.	Tian Dong	ཉེ་ཤིང་།	Liliaceae	Root tuber	Asparagus extract can reduce blood glucose level of diabetic mice [36, 37].	Asparagus extract
(12)	*Polygonum capitatum* Buch.	Tou Hualiao		Polygonaceae	Whole grass or aboveground part	Polygonum capitatum can improve the islet resistance of db/db mice [38].	
(13)	*Xanthium strumarium* L.	Cang Er	བྱིས་ཚེར།	The composite family	Fruit	The water extract of Fructus Xanthii has the effect of reducing blood glucose and maintaining blood glucose in hyperglycemic mice [39].	*Xanthium sibiricum* water extract
(14)	*Pseudognaphalium affine* (D. Don) Anderberg	Su Qucao	གར་བྷ་དྲ།	The composite family	Whole grass	The results showed that the total flavonoids of *Rhamnospermum* could effectively regulate the disorder of glucose and lipid metabolism in diabetic mice, and it can improve the antioxidant capacity of diabetic mice [40, 41].	Total flavonoids of *Rhamnospermum*
(15)	*Cyperus rotundus* L.	Xiang Fu	གླ་སྒང་	Cyperaceae	Rhizome	Total flavonoids of *Cyperus rotundus* have a good therapeutic effect on diabetic rats and can effectively reduce blood glucose, regulate blood lipid, and oxidative stress disorder [42].	Total flavonoids of Rhizoma Cyperi
(16)	*Apis cerana* Fabricius	Feng Mi	སྦྲང་མ།	Apidae	Honey	Honey can effectively promote the healing of diabetic foot ulcer, and wet compress of raw honey and Yunnan Baiyao can promote wound healing; and the curative effect is affirmative [43, 44].	
(17)	*Anthriscus sylvestris* (L.) Hoffm.	E Shen	ལྕ་བ	Umbelliferae	Root and leaf	The traditional Chinese medicine composition for the treatment of diabetic peripheral neuralgia provided by the invention has an affirmative curative effect on diabetic peripheral neuralgia [45].	
(18)	*Carthamus tinctorius* L.	Hong Hua	གུར་ཀུམ	The composite family	Flower	Safflower yellow injection can be used to treat early type 2 DR and improve the imbalance of VEGF and ES secretion [46]. Safflower injection has a positive effect on improving serum-related indicators and fundus blood flow in patients with DR, so its clinical application value in patients with DR is relatively higher [47]. Moreover, anthocyanin can reduce the contents of VEGF and PDGF in retina of diabetic rats through some mechanism, thus inhibiting the formation of new blood vessels and delaying DR [48, 49]. And safflower yellow applied to early patients is beneficial [50].	Safflower yellow pigment
(19)	*Punica granatum* L.	Shi Liu	སེ་འབྲུ།	Pomegranate family	Fruit	Upregulation of PDX-1 expression in pancreas and improvement of islet function in diabetic mice [51].	Pomegranate flower tanning polyphenols
(20)	*Fel Ursi*	Xiong Dan	དོམ་མཁྲིས	Ursidae	Gallbladder	The bear bile drainage solution exerts its antioxidant capacity to reduce the oxidative damage of nerve tissue mitochondria caused by DM [14]. UDCA eliminates no and oxygen-free radicals produced by STZ, protects *β*-islet cells from excessive apoptosis, and thus reduces blood glucose [52].	Ursodeoxycholic acid
